# Targeted temperature management in obstetrics for the prevention of perinatal encephalopathy

**DOI:** 10.55730/1300-0144.5859

**Published:** 2024-07-21

**Authors:** Aleksandr URAKOV, Natalya URAKOVA

**Affiliations:** 1 Department of General and Clinical Pharmacology, Faculty of Medicine, Izhevsk State Medical University, Izhevsk, Russia; 2 Department of Obstetrics and Gynecology, Faculty of Medicine, Izhevsk State Medical University, Izhevsk, Russia

**Keywords:** Hypoxic-ischemic encephalopathy, therapeutic hypothermia, targeted temperature management, stillbirth

**To the Editor**,

We read the article titled “Neurodevelopmental evaluation of newborns who underwent hypothermia with a diagnosis of hypoxic ischemic encephalopathy based on the Bayley-III scale,” written by Deveci et al. and published in *Turkish Journal of Medical Sciences*, with great interest [1]. We appreciated this work and would like to note what fruitful efforts the authors made while writing the article. The use of therapeutic hypothermia in obstetrics and gynecology is increasing every year as hypoxic-ischemic brain cell damage remains an important factor in stillbirth and neonatal encephalopathy in all countries of the world. To date, however, there is nothing more effective than therapeutic hypothermia. We agree that one significant factor in the efficacy of therapeutic hypothermia for neonatal health is its early application [1]. However, we would like to note that the aforementioned article does not consider the body temperatures of pregnant women. The temperature of the fetal head is completely dependent on the body temperature of the mother. Therefore, the intensity of aerobic metabolism and the oxygen demand of the fetal brain directly depend on the maternal body temperature [2–4]. One important factor of maternal body temperature is the time of day, as there are diurnal rhythms of body temperature [5]. Accordingly, the body temperature of the mother and fetus may be about 36.1 °C in the early morning and about 37.2 °C in the evening.

In other words, maternal and fetal temperatures can differ by 1.1 °C between morning and evening. According to the Arrhenius law, an increase in temperature by 10 °C increases the rate of chemical reactions by two times (i.e., by 100%), and so an increase in fetal body temperature by 1 °C increases the intensity of metabolism by an average of 10%. Therefore, the same reserve of fetal adaptation to hypoxia may have other significance for preserving the health and life of the neonate during vaginal delivery in cases of different body temperatures of the mother and the fetus. It is most likely that such a difference in temperature would occur in vaginal deliveries in the early morning and evening [2]. Based on this, we believe that the temperature of the mother and fetus in vaginal delivery at different times of the day is an important factor in neonatal encephalopathy. It is very important to use early therapeutic hypothermia not only for the newborn but also for the fetus [6]. However, the latter is not possible without lowering the temperature of the woman in labor. There are currently limited treatment methods for obtaining the “right” temperature, the most advanced of which is targeted temperature management (TTM) [7,8]. Temperature prophylaxis of hypoxic-ischemic brain injury is still being modernized, and it is necessary to adjust our approaches to the use of TTM in order to improve the efficiency and safety of birth and prevent not only perinatal encephalopathy but also stillbirth.

Not applicable.
